# Characteristics of the Copper,Zinc Superoxide Dismutase of a Hadal Sea Cucumber (*Paelopatides* sp.) from the Mariana Trench

**DOI:** 10.3390/md16050169

**Published:** 2018-05-18

**Authors:** Yanan Li, Xue Kong, Jiawei Chen, Helu Liu, Haibin Zhang

**Affiliations:** 1 Institute of Deep-Sea Science and Engineering, Chinese Academy of Sciences, Sanya 572000, China; liyn@idsse.ac.cn (Y.L.); kongx@sidsse.ac.cn (X.K.); chenjw@sidsse.ac.cn (J.C.); heluliu@sidsse.ac.cn (H.L.); 2 College of Earth and Planetary Sciences, University of Chinese Academy of Sciences, Beijing 100039, China

**Keywords:** superoxide dismutase (SOD), *Paelopatides*, expression, pressure, Mariana Trench, deep sea, sea cucumber

## Abstract

Superoxide dismutases (SODs) are among the most important antioxidant enzymes and show great potential in preventing adverse effects during therapeutic trials. In the present study, cloning, expression, and characterization of a novel Cu,Zn superoxide dismutase (Ps-Cu,Zn-SOD) from a hadal sea cucumber (*Paelopatides* sp.) were reported. Phylogenetic analysis showed that Ps-Cu,Zn-SOD belonged to a class of intracellular SOD. Its *K*m and *V*max were 0.0258 ± 0.0048 mM and 925.1816 ± 28.0430 units/mg, respectively. The low *K*m value of this enzyme represents a high substrate affinity and can adapt to the low metabolic rate of deep sea organisms. The enzyme functioned from 0 °C to 80 °C with an optimal temperature of 40 °C. Moreover, the enzyme activity was maintained up to 87.12% at 5 °C. The enzyme was active at pH 4 to 12 with an optimal pH of 8.5. Furthermore, Ps-Cu,Zn-SOD tolerated high concentration of urea and GuHCl, resisted hydrolysis by proteases, and maintained stability at high pressure. All these features demonstrated that the deep sea Ps-Cu,Zn-SOD is a potential candidate for application to the biopharmaceutical field.

## 1. Introduction

Reactive oxygen species (ROS) are required for several cellular biological contents and for various physiologic functions [1]. However, the accumulation of these reactive molecules in organisms will cause serious damage to DNA, proteins, and other bio-macromolecules and result in cell death, which is thought to be the pathogenesis of various diseases [2,3]. Superoxide dismutases (SODs, EC 1.15.1.1) play an important protective role during this process by preventing excessive ROS from damaging the cells. The SODs catalyze the disproportionation of superoxide anion radicals (O_2_^−•^) in a two-step reaction that eventually generate molecular oxygen (O_2_) and hydrogen peroxide (H_2_O_2_) [4]. Eukaryotic SODs are classified into Cu,Zn-SOD and Mn-SOD according to their metal cofactors. Among these, Cu,Zn-SOD is widely distributed in eukaryotes and comprises approximately 90% of the total SOD [5,6]. Point-mutations and the activity loss of Cu,Zn-SOD are linked to several serious human diseases, such as familial amyotrophic lateral sclerosis (FALS), Parkinson’s disease, Alzheimer’s disease, dengue fever, and cancer [3]. Hence, highlighting its therapeutic potential in medical and biological engineering is important [7,8]. Recently, several clinical trials using SODs as intervention agents have demonstrated its promising therapeutic potential for the treatment of ulcerative colitis, vitiligo, and Peyronie’s disease [9,10,11]. In addition, supplementary SOD can be used to reduce inflammation and ROS-mediated damage induced by certain therapeutic drugs. An era of less expensive and high-productive SODs has been opened, owing to their promising application in the medical field [8]. Extraction from various organisms and fermentative production using genetically engineered bacteria are effective ways to obtain SODs. Superoxide dismutases with stable kinetics are highly desired in biological engineering.

The deep sea is an extreme environment with some specific characteristics, including dark, low temperature, high hydrostatic pressure, and a shortage of food. Enzymes in deep sea organisms must function under these circumstances. Consequently, some unique proteins with novel properties not occurring in any known terrestrial organisms may be discovered [12,13,14]. Moreover, enzymes with special characteristics, such as esterase and α-glucosidase, have been identified from deep sea and other extreme environments [15,16]. The SODs of organisms living in extreme environments (e.g., *Cryptococcus liquefaciens, Marinomonas* sp., and *Thermus thermophilus*) have also been investigated [17,18,19].

Here, we discovered a novel Cu,Zn-SOD from a hadal sea cucumber (*Paelopatides* sp.) captured from the depth of 6500 m in the Mariana Trench. Considering that SODs of deep sea holothurian have not yet been studied, we cloned, expressed, and characterized the novel Cu,Zn-SOD. Based on our knowledge, this is the first research to study the pressure sensitivity of SOD.

## 2. Results

### 2.1. Sequence Analysis

**Figure 1:**
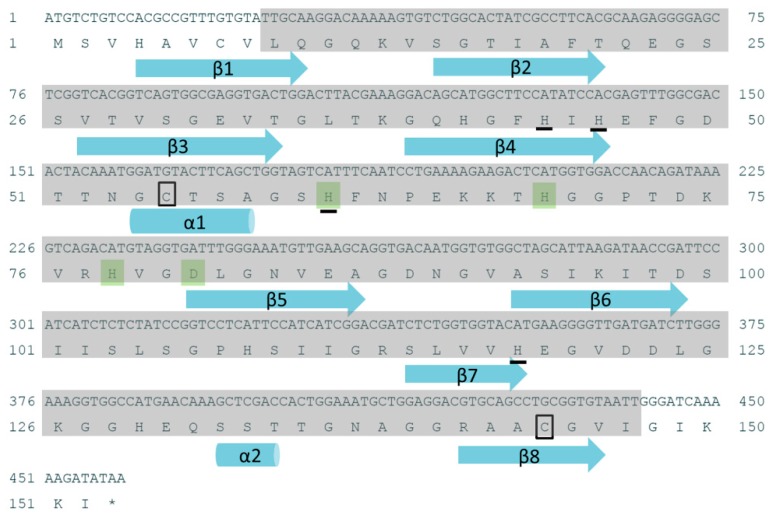
Nucleotide and deduced amino acid sequences of *Paelopatides* sp. copper, zinc superoxide dismutase (Ps-Cu,Zn-SOD). Conserved amino acid residues for Cu-binding are underlined, whereas residues for Zn-binding are shaded in green. Two cysteines predicted to form a disulfide frame are boxed. Cylinders and arrows represent helices and strands, respectively. The shaded part represents the predicted domain area.

The intact open reading frame (ORF) of the Ps-Cu,Zn-SOD gene was 459 bp long and encoded 152 putative amino acids. The SMART analysis showed that the important catalytic activity of Ps-Cu,Zn-SOD started from Leu-9 to Ile-147 (Figure 1). The calculated molecular weight and the theoretical pI of the deduced mature Ps-Cu,Zn-SOD was 15.40 kDa (recombinant protein was 18.23 kDa) and 6.53, respectively. Glycine (Gly) (17.8%) was the predominant amino acid, whereas methionine was the nadir (only 0.7%). The Ps-Cu,Zn-SOD did not contain any tryptophan and tyrosine residues. Based on the in vivo studies with *E. coli*, the estimated half-life of Ps-Cu,Zn-SOD was >10 h. The instability index was 21.39, indicating that the protein was stable. Moreover, no signal peptide or transmembrane domains were found, suggesting that Ps-Cu,Zn-SOD might be an intracellular SOD, and the phylogenetic analysis also confirmed this result.

**Figure 2:**
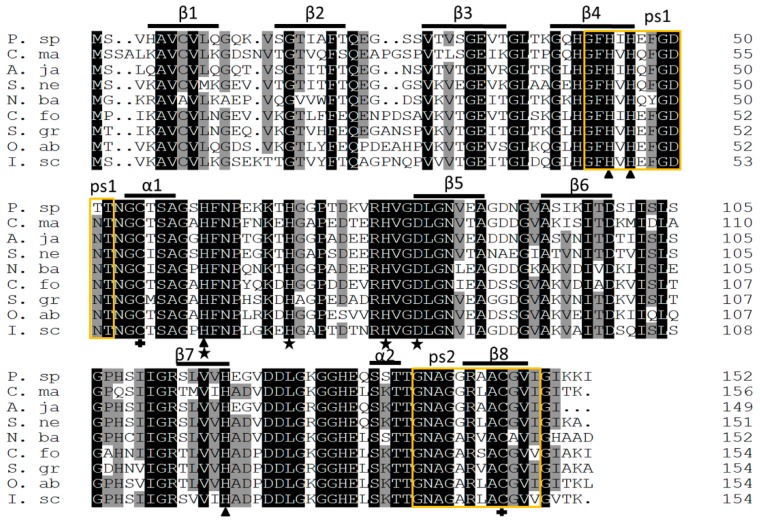
Multiple alignment of Ps-Cu,Zn-SOD with other Echinodermata and invertebrates. Identities and similarities are shaded in black and gray, respectively. The predicted eight β-strands, two α-helices, and two sequences of Cu,Zn-SOD family (yellow box, ps1 and ps2) are shown above the alignment. Amino acids for Cu- and Zn-binding are indicated with triangles and asterisks. The two cysteines forming a disulfide bond are indicated with plus signs. Information on the species used for the alignment is given in Table 1.

**Figure 3:**
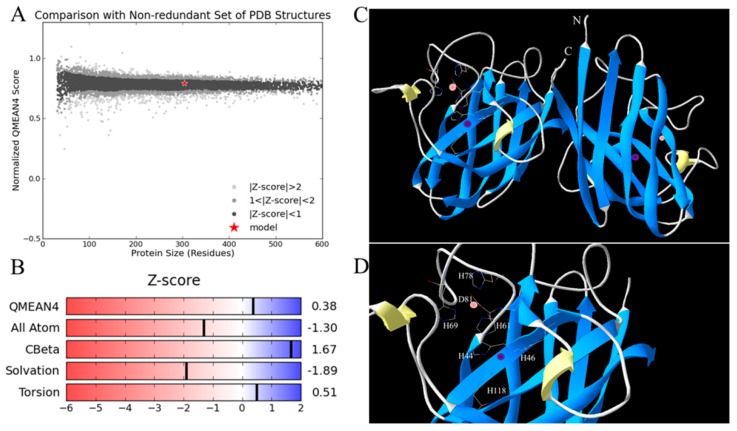
(**A**) Comparison of predicted structure with non-redundant set of PDB (Protein Data Bank) structures. The large black dots represent QMEAN Z-score between −1 and 1. The red star represents the model. (**B**) The four individual terms of the global QMEAN quality scores. (**C**) Predicted 3D structure of Ps-Cu,Zn-SOD using the Swiss model. Pink and purple spheres represent zinc and copper ions, respectively. The protein is a homodimer. N and C represent the N- and C-terminals of the polypeptide chain. (**D**) Close-up of the active sites.

A motif scan found two highly conserved Cu,Zn-SOD signatures in the deduced Ps-Cu,Zn-SOD amino acid sequence. Signature 1 (Accession: PS00087) with the pattern of [GA]-[IMFAT]-H-[LIVF]-H-x-[GP]-[SDG]-x-[STAGDE] began from Gly-42 to Thr (Threonine)-52 (GFHIHEFGDTT) and signature 2 (Accession: PS00332) with the pattern of G-[GNHD]-[SGA]-[GR]-x-R-x-[SGAWRV]-C-x(2)-[IV] spanned from Gly-136 to Ile-147 (GNAGGRAACGVI) (yellow box in Figure 2). Conserved copper (His (Histidine)-44, His-46, His-61, His-118) and zinc ion (His-61, His-69, His-78, Asp (Asparticacid)-81) binding sites and two cysteines (Cys (Cystine) -55, Cys-144) that formed a disulfide bond were found in each subunit of Ps-Cu,Zn-SOD. Furthermore, the predicted secondary structure of Ps-Cu,Zn-SOD contained eight β-strands and two α-helices (Figure 1). The predicted 3D model of the Ps-Cu,Zn-SOD was constructed by the Swiss-model server using the x-ray template of *Bombyx mori*’s Cu,Zn-SOD (PDB ID: 3L9E) [20]. They shared a sequence similarity of 52% and identity of 72.19%. The 3D model showed that Ps-Cu,Zn-SOD existed as a homodimer, and each subunit contained one copper ion and one zinc ion. The QMEAN4 Z-score of the 3D model of Ps-Cu,Zn-SOD was 0.38, and the GMQE was 0.90. The QMEAN consists of four individual terms, which are as follows: the interaction potential between C-beta atoms only, all atoms, the solvation potential, and the torsion angle potential, scoring 1.67, −1.30, −1.89, and 0.51, respectively (Figure 3A–C).

### 2.2. Homology and Phylogenetic Analysis

Pairwise alignment with other invertebrates indicated that the deduced amino acid sequence of Ps-Cu,Zn-SOD shared the highest identity and similarity with other Echinodermata homologs, such as *Apostichopus japonicus* (82.9% and 88.8%) and *Sterechinus neumayeri* (73.7% and 83.6%), and followed by *Ixodes scapularis* (69.7% and 79.4%), *Coptotermes formosanus* (68.8% and 80.5%), *Orussus abietinus* (68.4% and 77.4%), *Schistocerca gregaria* (67.5% and 78.6%), *Crassostrea madrasensis* (67.5% and 78.3%), and *Neoseiulus barkeri* (67.3% and 77.1%). The active sites of Ps-Cu,Zn-SOD were highly conserved among other examined orthologs, suggesting that the catalytic function of Cu,Zn-SOD was highly similar and conserved among different species (Table 1 and Figure 2).

**Figure 4:**
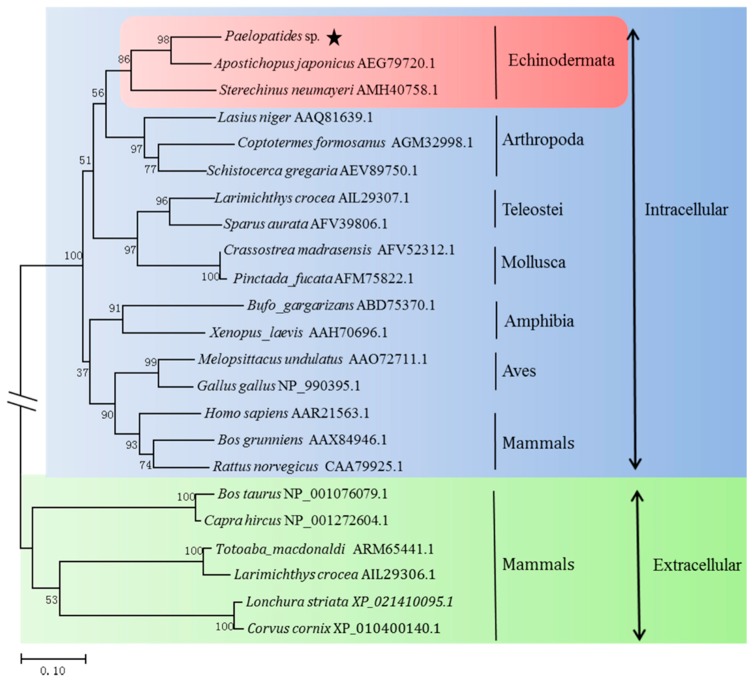
Neighbor-joining phylogenetic tree of Ps-Cu,Zn-SOD and Cu,Zn-SOD amino acid sequences from different species. The tree was built using MEGA7 and bootstrap was set as 1000. The star sign represents the position of Ps-Cu,Zn-SOD in the phylogenetic tree.

The phylogenetic tree was divided into two branches: intracellular Cu,Zn-SOD and extracellular Cu,Zn-SOD. The Ps-Cu,Zn-SOD belonged to the intracellular type as a monophyletic taxon and had the closest relationship with *Apostichopus japonicus* (98%). The Ps-Cu,Zn-SOD formed a branch with other Echinodermata and then formed a sister cluster with Arthropoda. Additionally, intracellular Cu,Zn-SOD of Echinodermata had a closer relationship with Teleotei and Mollusca, but was far from most vertebrates. Interestingly, the SOD sequences of the same taxonomic group clustered together and were in accordance with conventional taxonomy (Figure 4).

### 2.3. Expression, Purification, and Validation of the Recombinant Ps-Cu,Zn-SOD

**Figure 5:**
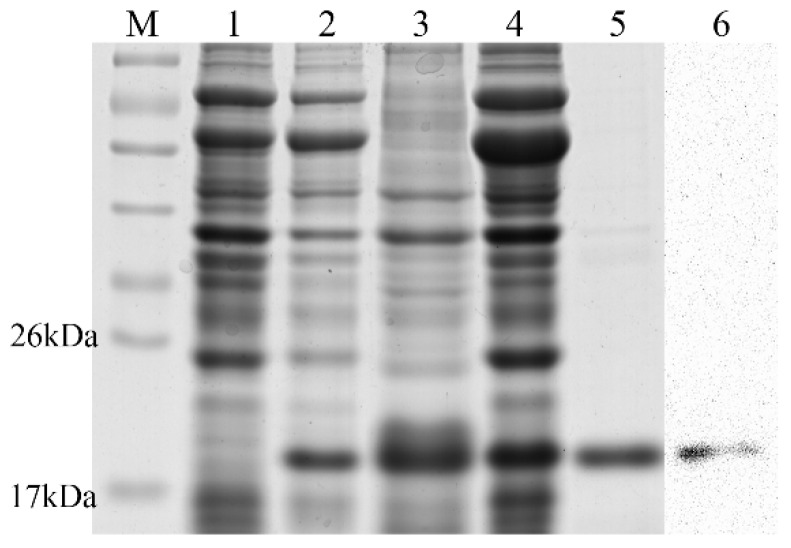
Analysis of the expressed protein via SDS-PAGE. M: protein marker, Lane 1: total proteins from *E. coli* pG-KJE8/BL21 containing recombinant plasmid before IPTG induction, Lane 2: total proteins from *E. coli* pG-KJE8/BL21 containing recombinant plasmid after IPTG induction, Lane 3: inclusion body after ultrasonication, Lane 4: supernatant after ultrasonication, Lane 5: recombinant protein purified using Ni^2+^-NTA column, Lane 6: Western blot of recombinant protein.

Analysis of the SDS-PAGE revealed that recombinant Ps-Cu,Zn-SOD was induced by 0.1 mM ITPG compared with the control (Figure 5, lanes 1 and 2) and exhibited a soluble expression in the supernatant (Figure 5, lane 4). The molecular mass of the recombinant protein was ~19 kDa, which was consistent with the estimated size of 18.23 kDa (Figure 5, lane 5). The protein yield was 1.91 mg/L culture. Protein purity was more than 95% (Figure 5, lane 5). The protein bands were then confirmed by Western blot analysis and all data indicated that Ps-Cu,Zn-SOD was successfully expressed and purified.

### 2.4. Effect of Temperature on Enzyme Activity

**Figure 6:**
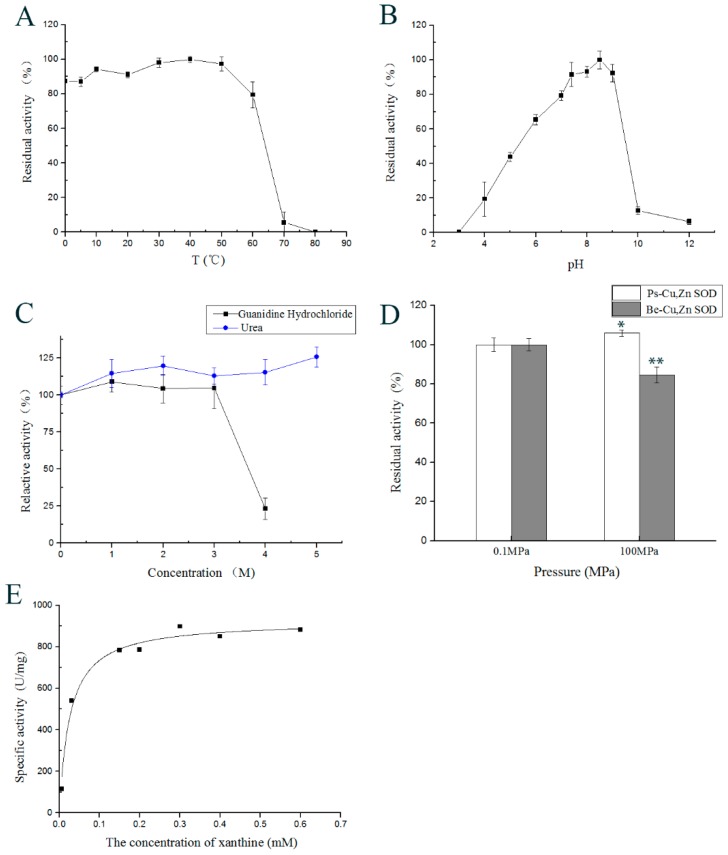
(**A**). Effect of temperature ranging from 0–80 °C on the enzyme activity. Each square in the graph represents the mean ± SD (*n* = 3). (**B**) Effect of pH on the enzyme activity. Tests were performed using different pH buffers ranging from 3.0–12.0 °C at 25 °C for 1 h. Each square in the graph represents the mean ± SD (*n* = 3). (**C**) Effects of denaturants on SOD activity. Values are mean ± SD of three separate replicates. The residual enzyme activity of the sample without denaturant was defined as 100%. (**D**) Effects of high hydrostatic pressure on SOD activity. Enzyme activity at 0.1 MPa at 5 °C was considered as 100%. Data are shown as mean values (*n* = 3) ± SD. (**E**) Michaelis-Menten equation of recombinant Ps-Cu,Zn-SOD. * *p* < 0.05; ** *p* < 0.01.

As shown in Figure 6A, a residual enzyme activity of >75% was maintained from 0 °C to 60 °C. Enzyme activity sharply dropped to 5.49% at 70 °C. A complete inactivation of the enzyme was achieved at 80 °C. The activity was maximal at 40 °C and was maintained at 87.12% at 5 °C. These results indicated that Ps-Cu,Zn-SOD had high activity at low temperature and was sensitive to high temperature.

### 2.5. Effect of pH on the Enzyme Activity

As shown in Figure 6B, Ps-Cu,Zn-SOD was active from pH 4.0 to 12 and demonstrated peak activity at pH 8.5. The majority of its activity was maintained at pH 5 to 9. The Ps-Cu,Zn-SOD was inactive at pH 3.0 and was activated by increasing the pH up to 8.5. After this point, enzyme activity sharply dropped with a further increase in pH. The enzyme retained >60% of its activity after 1 h incubation at pH 6 to 9, but it retained only 43.78% and 12.63% activity after incubation at pH 5 and 12, respectively. These findings suggested that an alkaline environment was more damaging to the enzyme’s structure than acidic environments.

### 2.6. Effect of Divalent Metal Ions on Enzyme Activity

The final concentrations of ions were set at 0.1 and 1 mM, and the results are shown in Table 2. Enzyme activity was inhibited by Mn^2+^, Co^2+^, and Ni^2+^, and the inhibition was strengthened with increasing ion concentration. Minimal effect on enzyme activity was noted with Ba^2+^, Mg^2+^, and Ca^2+^ at the stated concentrations. Significant inhibition of enzyme activity was observed at 1 mM of Cu^2+^ ions, whereas minimal effect was noted at 0.1 mM. In contrast, Zn^2+^ exhibited a positive influence on enzyme activity at both concentrations. The Mn^2+^ ion showed the maximal inhibition with a residual activity of 13.59 ± 2.04% at 1 mM concentration.

### 2.7. Effects of Inhibitors, Reductants, Detergents, and Denaturants on Enzyme Activity

Sensitivities of Ps-Cu,Zn-SOD to different inhibitors, reductants, and detergents are shown in Table 3. The enzyme activities were inhibited by DTT and EDTA and increasing the concentration enhanced the inhibition. However, >50% of the residual activities was retained after treatment with 10 mM EDTA and DTT for 40 min. The β-ME showed a positive effect on recombinant protein activity at both concentrations. At 1 mM β-ME, the enzyme activity increased to 122.70 ± 1.40%. The effects of detergent on Ps-Cu,Zn-SOD activity were different. The enzyme activity was strongly suppressed by SDS and sharply decreased to 37.01 ± 3.02% by 1% SDS. With detergents Tween 20, Triton X-100, and Chaps, the protein activities were slightly affected at 1% concentration and were enhanced at 0.1% concentration.

Using urea and guanidine hydrochloride as denaturants, the activities were determined after incubation at a series of concentration gradients for 1 h at 25 °C (Figure 6C). The enzyme was stable after treatment in 5 M urea and 3 M guanidine hydrochloride. However, the SOD was inactivated by 8 M urea (data not shown), and the activity sharply decreased in 4 M guanidine hydrochloride.

### 2.8. Resistance of Ps-Cu,Zn-SOD to Cleavage by Digestive Enzyme

To test the stability of the recombinant protein in digestive fluid, we performed a digestion experiment by measuring the residual enzyme activity after incubating for 0–3 h in digestive enzymes (trypsin/chymotrypsin complex 2400:400). Putatively, Ps-Cu,Zn-SOD sequence contained 12 trypsin and 4 chymotrypsin-high specificity cleavage sites, respectively (Table 4). However, the enzyme resisted digestion by trypsin and chymotrypsin complex even at a high enzyme/substrate (*w*/*w*) ratio of 1/77 for 3 h, and the residual activity was 93% (Table 5).

### 2.9. Effect of High Hydrostatic Pressure on Ps-Cu,Zn-SOD Activity

The result of high hydrostatic pressure assay is shown in Figure 6D. Commercial eukaryotic SOD from bovine erythrocytes was used for comparison under the same experimental conditions. The Ps-Cu,Zn-SOD maintained full activity under 100 MPa hydrostatic pressure. In contrast, SOD from bovine erythrocytes (Be-Cu,Zn-SOD) maintained only 84% activity at 0.1 MPa. These results suggested that Ps-Cu,Zn-SOD was insensitive to high hydrostatic pressure.

### 2.10. Kinetic Parameters of Ps-Cu,Zn-SOD

The recombinant Ps-Cu,Zn-SOD formed a dimer with a molecular weight of 38 kDa. Different concentrations of xanthine (0.006–0.6 mM) were used to determine the kinetic parameters of Ps-Cu,Zn-SOD. The *K*_m_ and *V*_max_ values of the recombinant Ps-Cu,Zn-SOD were 0.0258 ± 0.0048 mM and 925.1816 ± 28.0430 U/mg, respectively, based on the Michaelis–Menten equation at 37 °C and at pH 8.2 (Figure 6E). The R^2^ value of the curve fitting was 0.9775.

## 3. Discussion

Extreme organisms are the sources of novel enzymes. With the development of full ocean depth investigations, increasing numbers of organisms were found in abyssal living conditions. In the present study, a novel Cu,Zn-SOD from the hadal sea cucumber *(Paelopatides* sp.) and its enzyme kinetics under various parameters are reported.

The Ps-Cu,Zn-SOD is an oligomeric β-sheet protein that exhibits structural superiority toward kinetic stability [21]. For example, SOD from *Curcuma aromatic* maintained about 40% activities when EDTA reached 5 mM, maintained <40% activities when DTT reached 5 mM, and activities dropped to <60% when β-ME reached 6 mM [22]. SOD from the deep sea thermophile, *Geobacillus* sp. EPT3, was also significantly affected by EDTA and SDS, the activities of which dropped to <40% when EDTA reached 10 mM and were inactive when SDS reached 1% [23]. In contrast, the present SOD is more stable in these chemicals. Moreover, the CHAPS, Triton X-100, and Tween 20 exert a mild effect on proteins, which retain their conformation, and even enhance their solubility at low concentration (0.1%). These detergents have a significant impact on the activities of SOD from *Geobacillus* sp. EPT3 under 1% (*v*/*v*) concentration [23]. However, Ps-Cu,Zn-SOD could still maintain its original activity under 1% (*v*/*v*) concentration. Furthermore, Ps-Cu,Zn-SOD possesses remarkable resistance to hydrolysis of protease and high concentration of denaturants, as exemplified by maintaining original activity levels in 5 M urea and 3 M GuHCl. Similar results were also found in the Fe-SOD of *Sulfolobus acidocaldarius* and Cu,Zn-SOD of *Curcuma aromatica* [22,24]. These characteristics might be due to the conformational lock of the enzyme, which protects the important ligands and amino acids and maintains the catalytic active site, including the disulfide bonds [22]. The spatial structure of the Ps-Cu,Zn-SOD causes an unusually high unfolding energy barrier, resulting in slow unfolding rates in SDS, denaturants, and proteolytic enzymes.

In the present study, Ps-Cu,Zn-SOD displayed a low *K*_m_ compared to the other reported SODs. As a constant characteristic of enzymes, *K*_m_ reflects the affinity of the enzyme-substrate. Low *K*_m_ value suggests a high affinity for substrates even at low concentration and a high catalytic rate [22]. The *K*_m_ value of Ps-Cu,Zn-SOD was lower than many reported corresponding enzymes. For example, Cu,Zn-SOD from *Crocodylus siamensis* possess a *K*_m_ value of 6.075 mM for xanthine [25]. The strong enzyme-substrate affinity and high catalytic rate of Ps-Cu,Zn-SOD (*V*_max_ 925.1816 ± 28.0430 U/mg) make it a potential candidate for biological engineering [26].

Some researchers studied structure, mechanism, stability, and the biochemical characteristics of SODs from deep sea yeast, worm, fishes, and bacteria [17,27,28,29]. However, the effects of high hydrostatic pressure on SODs have yet to be studied. We performed the first high pressure tolerance experiment on SOD. As expected, Ps-Cu,Zn-SOD was stable under 100 MPa pressure. According to the sequences of Ps-Cu,Zn-SOD and Be-Cu,Zn-SOD, they share 64% identity and Ps-Cu,Zn-SOD contains slightly less proline (2.0%) than Be-Cu,Zn SOD (3.9%). Proline can break a helix and increase protein flexibility, which causes high compressibility and enhances the instability of the protein under high pressure condition [30]. However, Kawano compared the RNA polymerase amino acid sequence of *Shewanella violacea* (piezophilic deep sea bacterium) and *Escherichia coli* and suggested that protein flexibility might not be a determining factor in piezotolerance [31]. However, some critical amino acid residues in the domain and dimer interface of SOD could be responsible for the piezotolerance based on the structural discrepancy between Ps-Cu,Zn-SOD and any known normal Cu,Zn-SOD.

This work provides a novel and kinetically stable SOD from the hadal sea cucumber, which possesses special characteristics and has potential for medicinal and biological engineering applications.

## 4. Materials and Methods

### 4.1. Material, RNA Extraction, and cDNA Cloning

The hadal sea cucumber (*Paelopatides* sp.) was collected at a depth of 6500 m in the Mariana Trench (10°57.1693′ N 141°56.1719′ E), and the tissue was preserved. Total RNA was extracted using RNeasy Plus universal kits (Qiagen, Hilden, Germany) according to the manufacturer’s instruction. Quantity and purity of the total RNA were determined using NanoDrop 2000 ultramicrospectrophotometer (A260/A280 and A260/A230, Thermo Fisher Scientific, Waltham, MA, USA) and via electrophoresis on 1.5% agarose gel, respectively. Total RNA was sent to Novogene (Tianjin, China) for high-throughput sequencing, assembly, and annotation. Reverse transcription from total RNA to cDNA was performed using Takara reverse transcription kit (PrimeScript™ II 1st strand cDNA synthesis kit, Takara, Japan). The Cu,Zn-SOD sequences of Holothuroidea selected from GenBank were replicated to the *Paelopatides* sp. transcriptome database to prepare a local blast using the Bioedit software (Version7.0.5.3 Tom Hall, Carlsbad, CA, USA) with an *E*-value of 10^−90^. The Cu,Zn-SOD cDNA sequence of *Paelopatides* sp. with complete ORF and protein structure domain (analyzed on https://www.ncbi.nlm.nih.gov/orffinder/) could be used for designing special primers and PCR (ABI Inc., Austin, TX, USA) amplifications for subsequent steps. The intact ORF of Ps-Cu,Zn-SOD are available in the GenBank database with the accession numbers of MG989672.

### 4.2. Construction of Expression Vector

The primers F: CGGGATCCATGTCTGTCCACGCCGTTTGTGTATT and R: AACTGCAGTATCTTTTTGATCCCAATTACA with *BamH* І and *Pst* І sites (underlined) were designed using Primer 5.0 (Premier Inc., Palo Alto, CA, USA) to amplify the ORF of Ps-Cu,Zn-SOD. The PCR amplification was performed using PrimeSTAR^®^ GXL DNA Polymerase (Takara, Japan) in a 50 μl reaction system, according to the manufacturer’s instruction. The PCR condition included three steps as follows: 95 °C for 10 s, 55 °C for 15 s, and 68 °C for 10 s, for 30 cycles. Target PCR product was gel-purified, cloned into pMD18-T vector, and transformed into competent *E. coli* DH5α (Takara, Japan). Positive clones on ampicillin-containing LB plates were verified by sequencing with M13F(−47): CGCCAGGGTTTTCCCAGTCACGAC and M13R(−48): AGCGGATAACAATTTCACACAGGA universal primers. The single clone with the correct amplified sequence was propagated and extracted for plasmids. The plasmids, including Ps-Cu,Zn-SOD intact ORF sequence and cold-shock expression vector pCold II (Takara, Japan) were digested with restriction enzymes *BamH* І and *Pst* І based on the manufacturer’s protocol (Takara, Japan). The digested products from the plasmids and vector were ligated using T4 DNA ligase (Takara, Japan). The recombinant plasmids, pCold ІІ Ps-Cu,Zn-SOD, which contained 6× His-tag coding sequences at the 5′-end of the Ps-Cu,Zn-SOD gene were transformed into competent *E. coli* DH5α. Positive clones on ampicillin-containing LB plates were sequenced using pCold vector universal primers, pCold F: ACGCCATATCGCCGAAAGG and pCold R: GGCAGGGATCTTAGATTCTG.

### 4.3. Expression and Purification of SOD Protein

The recombinant plasmids, pCold ІІ Ps-Cu,Zn-SOD, were expressed in *E. coli* chaperone competent cells pG-KJE8/BL21 (Takara, Japan) under IPTG induction. The *E. coli* cells were grown in liquid LB medium containing 100 μg/mL ampicillin, 20 μg/mL chloramphenicol, 0.5 mg/mL l-arabinose, and 2 ng/mL tetracycline at 37 °C until the OD_600_ reached 0.4–0.6. The culture was placed on an ice-water mixture for 40 min, and then, IPTG was added (final concentration of 0.1 mM) to induce the expression of recombinant protein. The mixture was incubated at 15 °C overnight. The cells were harvested by centrifugation at 8000× *g*, 4 °C for 5 min. Pellet cells were washed twice with 1× PBS and then sonicated with an ice-cold binding buffer (50 mM Na_3_PO_4_, 300 mM NaCl, and 20 mM imidazole, pH 7.4) by using an ultrasonic cell disruptor until the solution became transparent. The lysate was centrifuged at 12,000× *g*, 4 °C for 20 min to collect the final supernatant with the recombinant protein.

The recombinant protein was purified using a 1 mL Ni^2+^-NTA column (Sangon Biotech, China). Ten resin volumes of binding buffer were used to equilibrate the column. Subsequently, the supernatant was added to the column. Next, the column was sequentially washed with 15 resin volumes each of binding buffer and wash buffer (50 mM Na_3_PO_4_, 300 mM NaCl, and 40 mM imidazole, pH 7.4). Finally, the target protein was eluted using 10 resin volumes of elution buffer (50 mM Na_3_PO_4_, 300 mM NaCl, and 300 mM imidazole, pH 7.4). The flow rate was controlled at 1 mL/min for every step. The collected target protein was transferred into a pretreated dialysis bag and dialyzed with 1× TBS at 4 °C. The dialysis solution was slowly stirred and replaced after 4 h, 8 h, and 14 h. Afterwards, the purified protein was concentrated to 1 mL by using a Millipore ultrafiltration tube (Burlington, MA,USA) Purity and expression condition of the recombinant protein were determined using 12% SDS-PAGE, followed by staining with Coomassie brilliant blue R-250.

### 4.4. Sequence Analysis, Structure Modeling and Phylogenetic Analysis

The intact ORF of Ps-Cu,Zn-SOD was translated into its amino acid sequence using the ExPASy translate tool (http://web.expasy.org/translate/). The physicochemical properties of the deduced Ps-Cu,Zn-SOD protein were predicted by the ExPASy ProtParam tool (http://web.expasy.org/protparam/). The conserved domain was searched using SMART (http://smart.embl-heidelberg.de/) and NCBI (http://www.ncbi.nlm.nih.gov/structure/cdd/). The signal peptide and the secondary structure were predicted using SignalP 4.1 Server (http://www.cbs.dtu.dk/services/SignalP/) and the Scratch Protein Predictor (http://scratch.proteomics.ics.uci.edu/), respectively. The motif sequences were identified by InterPro Scan (http://www.ebi.ac.uk/InterProScan/). A 3D homology model of Ps-Cu,Zn-SOD was constructed in a Swiss model server (http://swissmodel. expasy.org/) [32]. The quality of the 3D model was assessed using the QMEAN function. Generally, high QMEAN Z-scores indicate that the model has good quality and is close to the experimental structure [32]. Multiple sequence alignment was created using Clustal Omega (https://www.ebi.ac.uk/Tools/msa/). The phylogenetic tree of Ps-Cu,Zn-SOD was constructed using the amino acid sequences via the neighbor-joining method and was implemented in MEGA7 [33]. The reliability of the branching was tested with 1000 replicates.

### 4.5. Western Blot Analysis

The 6 × His recombinant protein was boiled at 100 °C for 5 min, centrifuged at 12,000× *g* for 1 min, separated by 12% SDS-PAGE gel, and then transferred to a PVDF membrane (Immobilon-membrane, Lot No. R6EA4496A, Millipore Inc., Burlington, MA, USA) via trans-blot SD semi-dry electrophoretic transfer cell (DYCP-40C, Beijingliuyi, China). The PVDF membrane was successively incubated with 5% BSA for 4 h, anti-6 × His antibody (primary antibody, diluted to 1:5000 with 5% BSA; ab18184, Abcam, Cambridge, UK) overnight at 4 °C and a secondary antibody (diluted to 1:10,000 with 5% BSA; ab6789, Abcam, Cambridge, UK) for 2 h. The PVDF membrane was washed with 1× TBST (50 mM Tris-HCl, 50 mM NaCl, and 0.05% Tween 20, pH 7.2) every time the incubation solution was changed. The target band on the PVDF membrane was dyed with Pierce™ ECL Plus Western blotting substrate (ThermoFisher, Waltham, MA, USA) according to the manufacturer’s instruction and detected using a chemiluminescent imaging system (ChemStudio, analytikjena, Jena, Germany).

### 4.6. Enzyme Characterization 

#### 4.6.1. SOD Assays and Protein Concentration Measurement

The SOD activity was determined via a spectrophotometric method using a SOD assay kit according to the manufacturer’s instruction (Nanjing Jiancheng Institute of Biology and Engineering, Code No. A001-1-1, Nanjing, China). Briefly, the assay was based on SOD’s ability to inhibit the oxidation of hydroxylamine in the xanthine-xanthine oxidase system. In all the assays described below, 1× TBS was used as the blanking solution and was tested under the same experimental conditions. Three replicates were prepared for each group. One unit of SOD activity was defined as the amount of enzyme that inhibited 50% of the hydroxylamine reduction [34]. The measurement of protein concentration was performed using a BCA protein assay kit (Sangon Biotech Company, Order NO. C503021, Shanghai, China).

#### 4.6.2. Thermostability of the Recombinant Ps-Cu,Zn-SOD

The thermostability of the recombinant Ps-Cu,Zn-SOD was assessed by quantifying residual activities after heat treatment. The protein samples were incubated at different temperatures (0 °C to 80 °C at an interval of 10 °C) for 20 min in 1× TBS, pH 7.4 [5]. After heating, the samples were immediately removed and placed on ice. The enzyme activities were determined according the method described above. The group with the maximum enzyme activity was considered as 100%, whereas the activity in other groups were accounted to the percentage of it.

#### 4.6.3. pH Stability of the Recombinant Ps-Cu,Zn-SOD

The pH stability of the recombinant Ps-Cu,Zn-SOD was determined by quantifying the residual activity as described above. The assay was performed according to the method of Zheng et al. [35] with slight modification. The buffer systems included 0.2 M citric acid-sodium citrate (pH 4.0 and 5.0), sodium phosphate buffer (pH 6.0, 7.0, 7.4, and 8.0), Tris-HCl (pH 8.5 and 9.0), and glycine-NaOH (pH 10.0 and 12.0). A series of different pH buffers were mixed with an equivalent volume of protein, and then incubated for 1 h at 25 °C. The residual activities were tested after incubation. The group with the maximal enzyme activity was considered to show 100%, whereas other groups showed the percentage of it.

#### 4.6.4. Effect of Ions on the Activity of the Recombinant Ps-Cu,Zn-SOD

The effects of divalent metal ions on the recombinant Ps-Cu,Zn-SOD’s activity were determined using the abovementioned method. Various metal salts (MgCl_2_, ZnCl_2_, BaCl_2_, CaCl_2_, CuCl_2_, NiSO_4_, CoSO_4_, and MnCl_2_) were mixed with the protein to a final ion concentration of 0.1 or 1 mmol/L. The mixtures were incubated at 25 °C for 40 min in 50 mmol/L Tris-HCl buffer (pH 7.5), and the residual activities were determined. The enzyme activity of the sample without metal ions was defined as 100%.

#### 4.6.5. Effects of Inhibitors, Reductants, Detergents, and Denaturants on the Activity of the Recombinant Ps-Cu,Zn-SOD

To test the resistance of Ps-Cu,Zn-SOD to inhibitors, reductants, detergents, and denaturants, we conducted the assays, as described by Zhu et al. [23] with slight modification. Ethylenediaminetetraacetic acid (EDTA) was set as an inhibitor, whereas β-mercaptoethanol (β-ME) and dithiothreitol (DTT) were types of reductants. Sodium dodecyl sulfate (SDS), 3-[(3-cholamidopropyl)dimethylammonio]-1-propane sulfonate (Chaps), Tween 20, and Triton X-100 were set as detergents. Urea and guanidine hydrochloride were set as denaturants. The enzyme was mixed with each reagent, incubated at 25 °C for 40 min in 50 mmol/L Tris-HCl buffer (pH 7.5), and then, the residual activities were measured. The incubation time with the denaturants was extended to 1 h. The mixture without additives was defined as 100%.

#### 4.6.6. Proteolytic Susceptibility

The proteolytic susceptibility assay of the recombinant Ps-Cu,Zn-SOD was performed as described by Chuianfu et al. [36] with minor changes. The recombinant Ps-Cu,Zn-SOD and proteolytic enzyme (trypsin/chymotrypsin complex 2400:400; Sangon Biotech order no. A606789, China) were mixed with a mass ratio of 1: 77 and incubated at pH 7.4, 37 °C for 0 h, 1 h, 2 h, and 3 h. The trypsin and chymotrypsin cleavage sites on the Ps-Cu,Zn-SOD sequence were deduced using a peptide cutter software (http://web.expasy.org/peptide_cutter/). Residual activities were analyzed, and the activity of the sample incubated for 0 h was defined as 100%.

#### 4.6.7. High-Hydrostatic Pressure Stability

The enzyme was placed in an injector sealed with parafilm and then incubated in a high hydrostatic pressure vessel at 100 MPa for 2 h at 5 °C. After decompression, the protein was placed on ice, and the residual activity was determined immediately. The entire process of compression and decompression was completed within 1 min. The enzyme activities at 0.1 MPa at 5 °C was considered as 100%. Bovine erythrocyte SOD (Beyotime, code no. S0088) was selected for comparison.

#### 4.6.8. Determination of Kinetic Parameters

Catalytic activity of the Ps-Cu,Zn-SOD was investigated using a series of xanthine (0.006–0.6 mM) working solutions via the determination method described above. The kinetic data (*K*m and *V*max) was obtained from the Michaelis-Menten equation by using nonlinear regression between the different substrate concentrations and enzyme activity.

### 4.7. Statistical Analysis

Statistical analysis was performed using SPSS 21.0 (IBM Company, Armonk, NY, USA). The Independent-Samples *T* test was used for the comparison between each of the two groups. *p* < 0.05 was considered to be statistically significant.

## Figures and Tables

**Table 1:** Species and GenBank accession numbers of organisms used in the homology analysis of Ps-Cu,Zn-SOD via pairwise alignment (https://www.ebi.ac.uk/Tools/psa/emboss_needle/).

Species	Genbank No.	Abbreviation	Size	Similarity %	Identity %	Gap %
*Apostichopus japonicus*	AEG79720.1	A. ja	149 aa	88.8	82.9	2.0
*Sterechinus neumayeri*	AMH40758.1	S. ne	151 aa	83.6	73.7	0.7
*Ixodes scapularis*	AAY66847.1	I. sc	154 aa	79.4	69.7	2.6
*Coptotermes formosanus*	AGM32998.1	C. fo	154 aa	80.5	68.8	1.3
*Orussus abietinus*	XP_012280251.1	O. ab	155 aa	77.4	68.4	1.9
*Schistocerca gregaria*	AEV89750.1	S. gr	154 aa	78.6	67.5	1.3
*Crassostrea madrasensis*	AFV52312.1	C. ma	156 aa	78.3	67.5	3.8
*Neoseiulus barkeri*	AON96404.1	N. ba	153 aa	77.1	67.3	0.7

**Table 2:** Effects of divalent metal ions on enzyme activity. Results are shown as mean (*n* = 3) ± SD. * *p* < 0.05; ** *p* < 0.01.

Divalent Metal Ions	Concentration/mmol·L^−1^	Relative Activity/%
Control	-	100 ± 3.94
Mn^2+^	0.1	96.86 ± 1.25 **
	1	13.59 ± 2.04 **
Co^2+^	0.1	93.54 ± 3.08 **
	1	42.33 ± 2.50 **
Ni^2+^	0.1	90.64 ± 4.28 **
	1	65.92 ± 3.78 **
Cu^2+^	0.1	103.13 ± 1.21
	1	13.97 ± 4.68 **
Ba^2+^	0.1	101.42 ± 1.59
	1	98.16 ± 2.76 *
Mg^2+^	0.1	100.21 ± 4.78
	1	99.42 ± 4.12
Ca^2+^	0.1	100.91 ± 3.29
	1	101.06 ± 2.08
Zn^2+^	0.1	114.07 ± 4.94 **
	1	111.87 ± 4.42 **

**Table 3:** Effect of inhibitors, detergents, and denaturants on enzyme activity. The results are shown as mean (*n* = 3) ± SD. * *p* < 0.05; ** *p* < 0.01.

Inhibitors, Detergents, and Denaturants	Concentration	Relative Activity/%
Control	-	100 ± 3.71
EDTA	1 mmol·L^−1^	80.86 ± 4.60 **
	10 mmol·L^−1^	71.86 ± 4.86 **
DTT	1 mmol·L^−1^	98.93 ± 0.81 *
	10 mmol·L^−1^	56.62 ± 4.27 **
β-ME	1 mmol·L^−1^	122.70 ± 1.40 **
	10 mmol·L^−1^	118.78 ± 2.99 **
Triton X-100	0.1%	106.66 ± 1.63 **
	1%	100.91 ± 1.70
SDS	0.1%	63.59 ± 4.30 **
	1%	37.01 ± 3.02 **
Chaps	0.1%	110.37 ± 3.13 **
	1%	108.32 ± 3.99 **
Tween 20	0.1%	107.19 ± 1.40 **
	1%	98.20 ± 1.58 *

**Table 4:** The prediction of cleavage site of Ps-Cu,Zn-SOD.

Name of Enzyme	No. of Cleavages	Positions of Cleavage Sites
Chymotrypsin-high specificity (C-term to [FYW], not before P)	4	20, 43, 48, and 62
Trypsin	12	13, 38, 66, 67, 75, 77, 96, 113, 126, 141, 150, and 151

**Table 5:** Cleavage effect of digestive enzyme on Ps-Cu,Zn-SOD at different time periods. The results are shown as mean (*n* = 3) ± SD. ** *p* < 0.01.

Time (h)	Relative Activity (%)
0	100 ± 4.30
1	101.09 ± 8.58
2	96.89 ± 3.98
3	93.36 ± 5.62 **
